# Mineral and Botanical Composition of Honey Produced in Chile’s Central-Southern Region

**DOI:** 10.3390/foods11030251

**Published:** 2022-01-18

**Authors:** Iris Lobos, Mariela Silva, Pablo Ulloa, Paula Pavez

**Affiliations:** 1 Centro Regional de Investigación Remehue, Instituto de Investigaciones Agropecuarias INIA, Osorno 5290000, Región de Los Lagos, Chile; msilva.lemus@gmail.com (M.S.); paulita2823@gmail.com (P.P.); 2 Centro Regional de Investigación La Platina, Instituto de Investigaciones Agropecuarias INIA, Santiago 8820000, Región Metropolitana, Chile; pablo.ulloa@inia.cl

**Keywords:** honey, quality parameters, mineral content, melissopalynological composition

## Abstract

The chemical composition and quality of honey depend on the floral and geographical origin, extraction techniques, and storage, resulting in a unique product for each area. Currently, consumers are not only concerned about the chemical composition, quality, and food safety of honey, but also about its origin. The objective of this study was to characterize honeys produced in Chile’s central-southern region from a mineral and botanical perspective, thus adding value through differentiation by origin. Two hundred honey samples were used and underwent analysis such as melissopalynological composition, nutritional composition, and color. Forty-seven melliferous floral species were identified, out of which 24 correspond to exotic species and 23 to native species. Fifty-six percent were classified as monofloral honeys, 2% as bifloral, and 42% as multifloral. Moisture mean values (17.88%), diastase activity (15.53 DN), hydroxymethylfurfural (2.58 mg/kg), protein (0.35%), and ash (0.25%) comply with the ranges established by both the national and the international legislation; standing out as honeys of great nutritional value, fresh, harvested under optimal maturity conditions, and absence fermentation. Regarding color, light amber was prevalent in most territories. The territory where honey was produced, denoted relevant differences in all the parameters studied.

## 1. Introduction

Honey is a natural sweetener with functional properties (antioxidant, antimicrobial, anti-parasitic) [1,2] and potential properties (anti-inflammatory, anti-hypertension, prebiotic, and probiotic effects) [3]. Its chemical composition and quality greatly depend on the floral source picked by the bees, climate conditions, humidity inside the hive, geographical origin, manipulation during extraction and storage, obtaining as a result a unique and specific product to each area. Moreover, honey’s botanical and geographical origin can be determined due to its high pollen content which is the product of the collecting or harvesting by bees from different species [4]; thus honeys be used as a biomonitor to identify the level of pollution of soil, water, and air [3,5].

Botanic origin of honey guarantees the geographic origin and widely affects its commercial value [6]. The preference of one type of honey over others in the market can also be influenced by its color and organoleptic characteristics [7]. In this sense, defining the different attributes of honeys becomes important for its commercialization. The international honey markets have intensified efforts to characterize and authenticate it, as this plays an important role for both for consumers and beekeepers. The FAO/WHO 2019 [8] stablished basic quality parameters for honey aimed for human consumption, which must be taken into consideration for commercialization—highlighting humidity, ashes, carbohydrates content, electrical conductivity, hydroxymethylfurfural, and diastase index. These parameters, in addition to being quality indicators, are also measures of honey freshness and allow to evaluate the management of the processing and storage [9].

Currently, consumers are not only concerned about the chemical composition, quality, and safety of honey, but also about its origin. In this sense, Chile is known for its extensive geography and diversity of climates. According to the data showcased in the Chilean biodiversity reports made by the Ministerio de Medio Ambiente [10] in 2017, around 51% of the total continental surface of the country was compound by grassland, scrubland, and native forest. All of the surface native forest corresponds to 19% of national land cover which is concentrated mainly in the southern regions of the country. Thus, beekeeping production can result in differentiated honeys.

Accordingly, the market demand for products differentiated by origin and the challenge of adding value to the beekeeping industry make evident the need to correlate the chemical composition of honeys from different regions with their geographical and botanical origin.

In Chile, there are studies for ulmo (*Eucrphya cordifolia*) and soapbark (*Quillaja Saponaria*) monofloral honeys which describe the pollen origin and the physicochemical parameters of the samples. However, those studies have been focused in the northern and central areas of the country [11,12,13,14]. The profile of volatile, non-volatile, and semivolatile compounds has been described along with antioxidant and antimicrobial activity in ulmo honeys in the southern area of the country (Puerto Varas) [15]. Overall, the number of samples analyzed by geographical area is usually low, which has hindered us from characterizing in depth the types of honeys produced in the extreme southern regions of our country—from the perspective of the development of beekeeping—such as the Los Lagos Region, which is made up by four provinces (provinces of Osorno, Llanquihue, Chiloé, and Palena (Patagonia Verde)). Due to the extension of this region, each of these provinces present very different climatic and botanical characteristics, except for Osorno and Llanquihue, which are geographically connected. Therefore, it is very important to obtain information about the quality and characteristics of these honeys.

It is also important to consider that the central-southern region of Chile has a great diversity of flora, made up of: native species (those that were originated or arrived naturally in the country, without human intervention); endemic species (those that inhabit in a natural way only in one zone, for example: a continent, a country or an island); and exotic species (non-native species of a region or country which arrived intentionally or accidentally, usually as a result of human activity) [16] which contribute and differentiate the honey production of the country.

The objective of this study was to characterize honeys produced in Chile’s central-southern region from a mineral and botanical perspective, thus adding value through differentiation by origin.

## 2. Materials and Methods

### 2.1. Sample Collection

**Figure 1:**
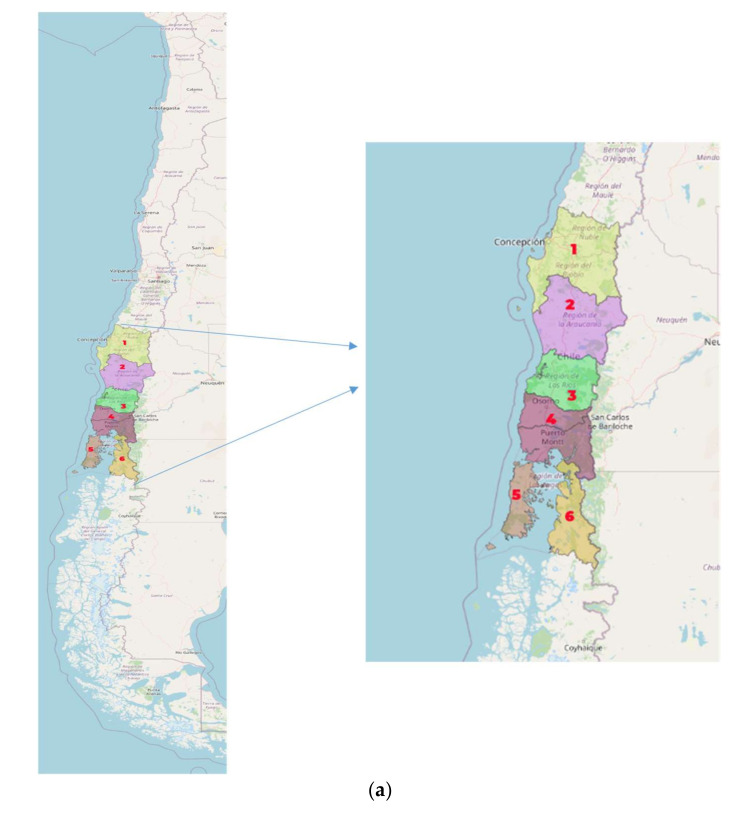

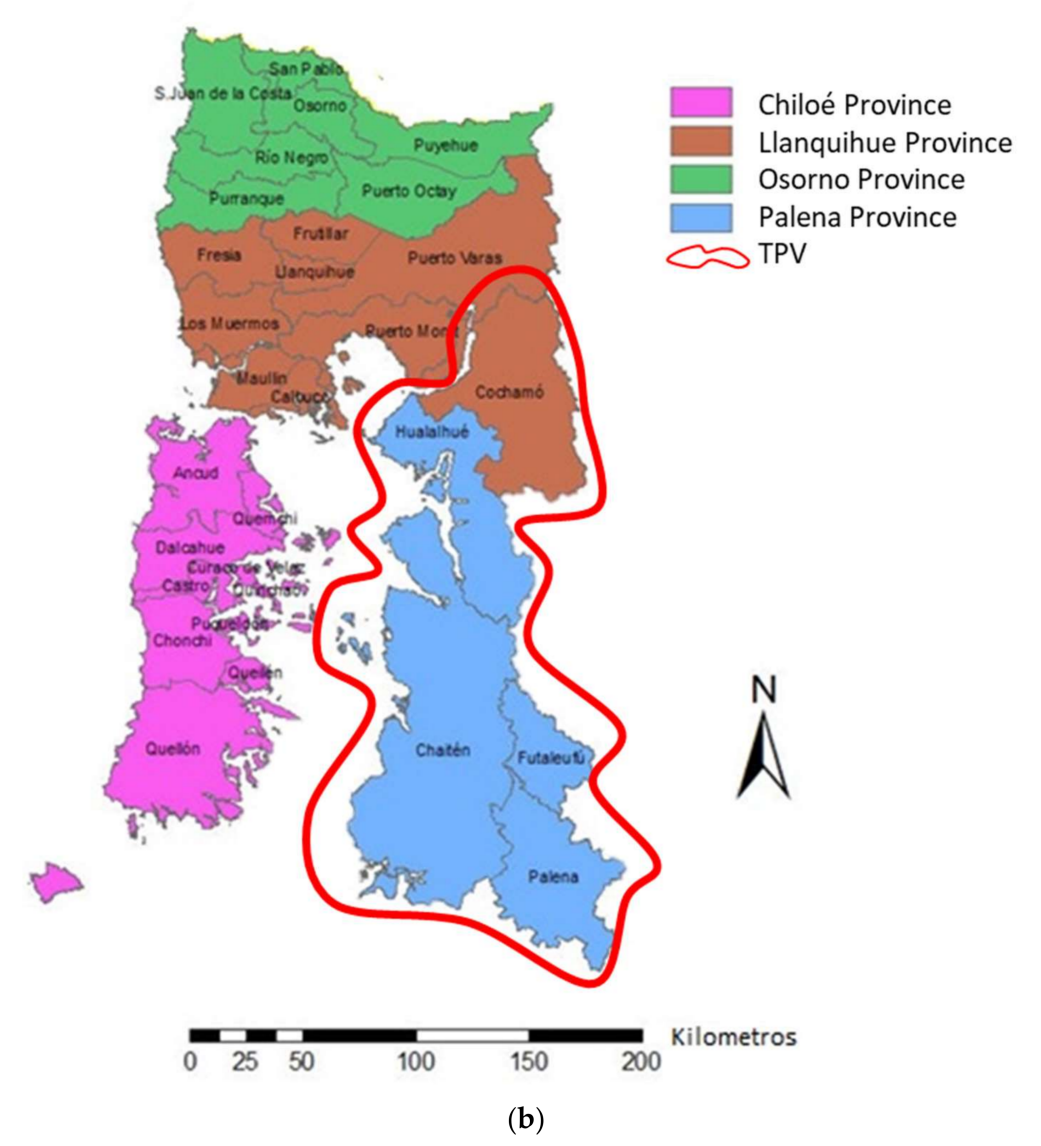
(**a**) Map of Chile (each number represents a sampling zone). (**b**) Political map of the Los Lagos region, showing the provinces. TVP is outlined in red, it includes the whole Palena province its four districts (Chaitén, Futaleufú, Hualaihué, and Palena) and the Cochamó district in the Llanquihue province.

A total of 200 honey samples from different districts and areas of Chile’s central-southern region were collected and analyzed (latitude 36°37′00″ south and longitud 71°57′00″ west to the north; up to latitude 43°40′56.64″ south and longitude 72°0′25.42″ west to the south) Figure 1a.

All samples were harvested during two seasons—March–April 2019 and March–April 2020—directly by the beekeepers. The honey samples were stored at room temperature until analysis. Table 1 shows the geographic location and number of samples by sector of harvest.

### 2.2. Melissopalynological Analysis

The botanical origin of honeys was determined by a melissopalynological analysis according to the Chilean Norm NCh2981:2005 [17]. Samples were processed and analyzed in the INIA Remehue laboratories (Osorno, Chile).

### 2.3. Physicochemical Parameters

The humidity content in honeys was determined according to the Chilean Norm NCh3026.Of2006 [18]. The Diastase Index was determined according to Schade (Harmonized Methods of the European Honey Commission) [19]. The hydroxymethylfurfural (HMF) content was determined according to the Chilean Norm NCh3046.Of2007 [20]. The protein content was determined using the Kjeldahl method, [21]. The ash content was determined by calcination [22,23] The macro and micro minerals were determined by atomic absorption spectrophotometry and atomic emission spectrophotometry [24].

### 2.4. Color Index

A honey color photometer (Hanna HI 96,785 HANNA Instruments, Chile) was used for color analysis. This equipment provides a value on the mm Pfund scale, through an optical system that used a tungsten lamp, a band filter, and a silicon photodetector. Table 2 shows the USDA universal color scale, which allows the classification of honey according to its color [25].

### 2.5. Statistical Analysis

InfoStat statistical software was used for data analysis. Shapiro–Wilk normality test was conducted. A statistical analysis was done using one-way ANOVA and a Tukey test for multiple comparisons of means with a significance level of *p* < 0.05 for the data with normal distribution; and a Kruskal–Wallis test was used for the data that did not present a normal distribution.

For the principal component analysis and cluster analysis, R and FactiMineR software packages were used. For clusters, Euclidean distance was used.

## 3. Results and Discussions

### 3.1. Melissopalynological Analysis

**Figure 2:**
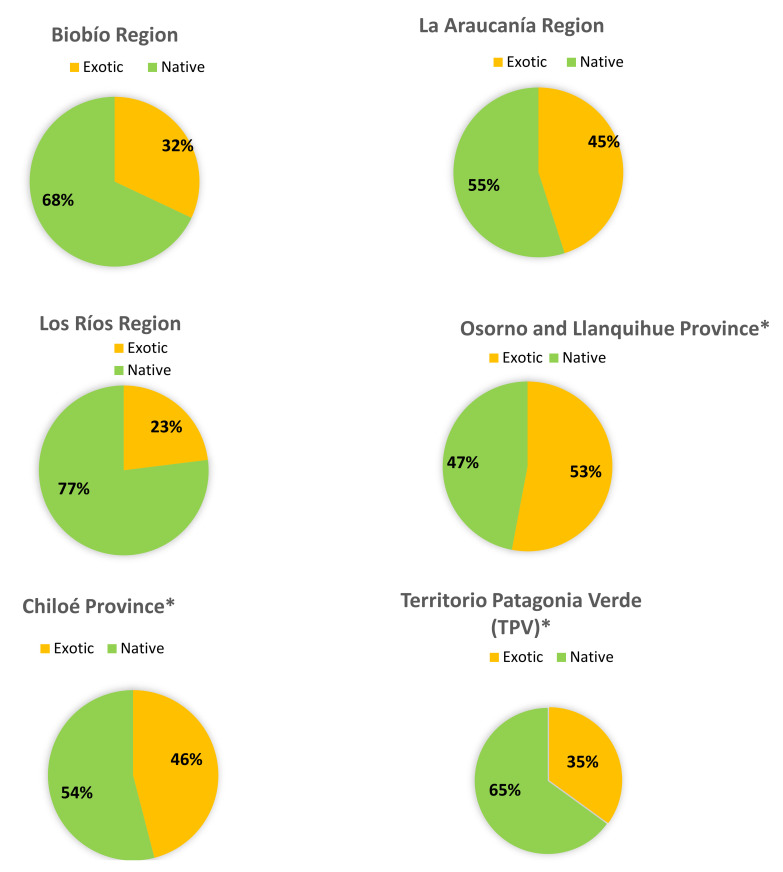
Distribution of native and exotic floral species found in honey samples according to the collection territory. * Territories belonging to the Los Lagos region.

The melissopalynological analysis allowed the identification of 47 melliferous floral species in the 200 honeys analyzed, out of which 24 correspond to exotic species and 23 to native species. Figure 2 shows that Los Ríos Region presented the highest percentage of native species in its honeys (77%), whereas the Osorno and Lanquihue province had the highest content of exotic species (53%). These results make evident the low anthropogenic intervention regarding the use of soils destined for forest, agriculture, and livestock activities. Since many of the sampled sectors still have limited access, several of these territories have been able to preserve their natural state.

Among the predominant native species in this study *Eucryphia cordifolia* (Ulmo) was found (Table 3); an evergreen tree, native to southern South America which possesses a honey-bearing flower highly sought after by bees due to the large amount of nectar it produces; and *Weinmannia trichosperma* (Tineo) a perennial tree endemic to the temperate rainforest [26]. Both species are widely distributed in Territorio Patagonia Verde (TPV), Osorno and Llanquihue Province, and Los Ríos Regions. These zones present important quantities of native species in protected nature reserves and species belonging to Valdivian Forest, characteristic of this southern zone [27].

On the other hand, in the La Araucanía and Biobío Regions, there are noteworthy species such as *Quillaja saponaria Mol.* (Soapbark), an endemic tree which grows in a wide range of habitats in the forests and scrublands of the Mediterranean Climatic zone of central Chile; and its nectar is known for producing one of the most emblematic honeys of the country [6].

Among the exotic species, Fabaceae (*Lotus* sp., *Melilotus* sp.)—herbaceous species that make up a large part of the grassland of Los Ríos and Los Lagos Region—predominated; concentrating specifically in the Osorno and Llanquihue Province, an area characterized by grassland-based animal production [28].

For the honey samples from Chiloé Island Province, it was possible to observe the presence of *Eucalyptus globulus* (Eucalyptus) (Table 3), a species that predominated in the samples collected in sectors of the northern part of the island, an area used for forestry production [29]. Species *Brassica rapa* (Field mustard), a very common wild plant throughout Chile [30], which grows as a weed in crop fields and along roadsides [31], was found mainly in Green Patagonia Territory.

On the other hand, exotic species that predominated was *Castanea sativa* (Sweet chestnut) a tree whose fruit plantations are in Biobío Region, and also in plantations for forestry use in the La Araucanía Region [32]. The Biobío and La Araucanía Regions also had *Rubus ulmifolius* (Elmleaf blackberry), species of fruit distributed from Coquimbo to Los Lagos Regions [33].

In addition, the Instituto Nacional de Normalización (INN) established Chilean Norm NCh2981:2005 [17] with the purpose of having a method that allows to differentiate the botanical origin of honeys produced in Chile, and at the same time classify them as: monofloral honeys (one species compound a 45% of the total pollen grains counted and identified in the sample); bifloral honey (conjointly two species compound more than 50% of the total pollen grains counted in the sample, and they do not present a difference of more than 5% between them); and multifloral honey (none of the species reach 45% of the total pollen grains counted, nor are there two species that dominate the pollen section).

In that sense, the total of honey samples analyzed (*n* = 200), 56% classified as monofloral honey being *Eucryphia cordifolia* (Ulmo) the main species found with a total of 55 samples (Table 4). The TPV was the territory that presented the largest amount of Ulmo monofloral honey (36.4%) followed by Los Ríos Region (23.6%) with 13 samples. The species that predominated in the Osorno and Llanquihue province—Los Lagos Region—were species of the legume family (*Lotus* sp. *y Melilotus* sp.) with 55.5%. The Los Ríos Region also stood out for presenting 63.6% of monofloral honeys of species *Weinmannia trichosperma* (Tineo). The Biobío Region was the only territory with monofloral honeys from the *Quillaja saponaria Mol.* (Soapbark) and with no presence of *Eucryphia cordifolia* (Ulmo) honey.

In Table 4, it can also be observed that only 2% of the analyzed honeys belong to the category of bifloral honeys being *Eucryphia cordifolia* (Ulmo), *Weinmannia trichosperma* (Tineo), and *Caldcluvia paniculata* (Tiaca) the predominant species. Lastly, 42% of honeys classified in the category of multifloral honeys (Table 4). These results correlate with the data obtained by [34], who described Chilean honey production, mostly Ulmo monofloral honey from the Los Lagos Region and exotic species such as *Rubus ulmifolius* (Elmleaf blackberry) in honey from the La Araucanía Region.

### 3.2. Physicochemical Parameters

The results indicate relevant differences in the chemical composition of the samples according to the production territory of honey for all the parameters that are being studied (Table 5). Moreover, it is possible to observe that the average values of moisture (17.88%), diastase activity (15.53 DN), hydroxymetylfurfural content (2.58 mg/kg), protein (0.35%), and ash (0.25%) comply with the values established in the Chilean legislation [35], for floral-origin honey according to the parameters mentioned (20% maximum moisture; diastase number >8; HMF < 40 mg/kg; and ash content <0.8%). Regarding color, there is a prevalence of light amber shade in most territories.

The moisture content varies between 17.4–18.4%; which indicates that honeys were at an optimum point of maturity [36]—i.e., they would not be affected by premature fermentation or crystallization processes; implying a conservation of physicochemical properties and quality of honey [37]. Furthermore, the moisture values presented in this current study are superior to those reported by [38], in multifloral honeys from southern Chile (15.84%) [39]; in honeys from the Biobío Region (16.28%) and [40] in honeys from the Chubut province Argentina. These differences could be related to fact that the moisture content in honeys is influenced by factors such as the botanical and geographical origin of the nectar, relative humidity, soil conditions, time of collection, degree of ripening, harvesting practices, extraction, processing, and storage conditions [41,42].

The hydroxymethylfurfural (HMF) is an unwanted compound (aldehyde) produced by sugar degradation in an acidic medium through Maillard reactions. Its formation occurs naturally through time, and this can be accelerated if honey is exposed to high temperatures in the process of extraction, homogenization, etc. [43]. HMF concentration in the honeys analyzed fluctuated between 1.0–3.8 mg/kg, being the Biobío Region the one that presented the highest concentration; this correlate with results obtained by [44] in honeys of the same region. This can be attributed to the climatic conditions in terms of the average temperatures, which gradually drop from La Araucanía to the Los Lagos Regions [40]. The values obtained in each territory are indicators of freshness and show the use of good beekeeping practices during honey harvesting.

Concerning the diastase enzyme—one of the most important enzymes that helps to maintain the balance of sugars in honey and to avoid crystallization—it was one of the parameters that presented more variations among territories, which could be explained because the botanical origin is one of the various factors affecting the activity of this enzyme; since the enzymatic activity that honey presents is, to a certain extent, given by the bees as it is also for the presence of pollen rests [44]. Although TPV had the lowest activity, 10.9 ± 5.1 DN among the territories (this territory presented two samples with values below 8), this value is above the established by the current legislation [35], indicating that the honeys studied are fresh and have not been subjected to excessive heat treatments [45].

The protein content of honey is strongly influenced by its pollen content—i.e., by the number of pollen grains per gram of honey and the diversity of pollen types contained in the honey [46]. The mean protein content obtained in this study (0.35%) is in the expected range for honeys of excellent quality (0.1–0.5%) [3]. This was one of the parameters that presented the least variation, which can be explained by the high percentage of monofloral honeys found (56%), which coincides with what was reported by [47,48]. Furthermore, the protein content obtained in the different territories show that the samples analyzed have not been adulterated, overheated, or stored for long periods of time [49]. Additionally, it is important to consider that—from a technological point of view—the presence of proteins can be unwanted since the surface tension is lower when increasing the protein content, leading to the presence of foaming and air bubbles [3].

Meanwhile, ash content in honey can be an indicator of environmental pollution [50] as well as a quality criterion to evaluate the botanical origin of the honey. The mean ash content obtained in the present study was low (0.25 ± 0.1%)—with no major variations between areas—and complies with the Chilean regulations in all the territories analyzed. Moreover, the concentrations obtained correlate with those reported by [39] for honeys harvested in the districts of Antuco and Nacimiento; [51] for honeys of floral origin (<0.6%); and [52] for honeys from Saudi Arabia and Kashmir (0.23 ± 0.02 and 0.30 ± 0.03%), respectively.

Color is one of the first characteristics observed and the optical property with the greatest variability. In beekeeping, it is one of the sensory properties attributed to quality, botanical origin, functional properties, soil type, and climate where the flowers—from which the nectar is collected—grow, among others [53]. The samples analyzed presented variations between territories with colors ranging from extra light amber (43.7 mm Pfund) to light amber (73.9 mm Pfund) (Table 5). The samples from the Chiloé territory were the lightest and exhibited significant differences with the other territories analyzed. The color difference observed in the current study may be related to the presence of *Eucalyptus globulus* (Eucalyptus) in the samples from the province of Chiloé, which was nonexistent in the other territories analyzed.

The resulting colors of honeys from Chile’s central-southern region—without considering the territory as a classification factor—were found in a range that varied between 22–101 mm on the Pfund scale, which corresponds to honeys ranging in color from white to amber. Of the total samples (*n* = 200) analyzed, 5.5% were classified as white, 25% as extra light amber, 65.5% as light amber, and 3% as amber.

Conversely, honeys from rapeseed, Tiaca and Fabaceae, classified as monofloral, presented the lowest ranges of color—46.8 and 54.0 mm Pfund, respectively—which correlates with what was reported by [54], where the grassland samples were the ones with the lightest shades compared to the other samples studied. Meanwhile, species such as *Weinmannia trichosperma* (Tineo) and *Quillaja saponaria Mol*. (Soapbark) were predominant in honeys with darker tones; similar results have been reported in *Quillaja Saponaria* honey samples from Libertador Bernardo O’Higgins Region, which presented values closer to 80 mm in the Pfund scale [55]. In addition, authors such as [56] who analyzed monofloral Eucalyptus honey, have stated that extra light amber honeys predominate in this type of honeys. It was also possible to observe that honeys classified as amber contained the highest potassium concentrations (1829.3 ppm), which correlates with the data reported by [57] in honeys produced in Serbia, where honeys that presented darker shades had a higher potassium and magnesium content.

On the other hand, honey is a good source of minerals, which are essential for the growth and health of consumers. Table 6 shows the mineral content of the analyzed honeys, where it is possible to observe average concentrations of 72.48 ppm P; 73.26 ppm Ca; 22.23 ppm Mg; 13.36 ppm Na; 1090.08 ppm K; 36.68 ppm Al; 0.9 ppm Zn; 2.28 ppm Mn; 3.70 ppm Fe; and 0.77 ppm Cu. Macro and micro minerals levels are within the ranges described by [58] (Table 6), with the exception of aluminum and copper, which are 1.5 and 0.8 times higher, respectively.

The minerals with higher concentrations were potassium (K) and sodium (Na), which is in accordance with what was reported by [59] in different honeys from the Malaysian Regions. In addition, the analyzed honeys presented lower concentrations of all the elements studied by said author.

A study conducted by [36] in honeys from different territories of Argentina reported lower contents than those reported in this study of potassium (816.2 ppm), calcium (10.86 ppm), copper (0.29 ppm), iron (2.99 ppm), and manganese (1.05 ppm); while sodium (33.19 ppm), zinc (1.17 ppm), and magnesium (22.64 ppm) had reported higher values.

The high values of Al and Cu may be due to anthropogenic factors [60]. In the case of aluminum content, authors—such as [29]—have stated that it could be associated with their geographical origin since they come from apiaries located in soils of volcanic origin. These soils are distributed between the Biobío and Los Lagos Region and are characterized by high free-aluminum contents.

The concentrations of aluminum found in this study are in average, 17 times higher than those reported by [61] and [13] in monofloral and multifloral honeys studied between the regions of Coquimbo and Los Lagos. This can be a consequence of the high presence of “trumao” soils, mostly in the Los Lagos and Los Ríos Regions (regions that are characterized for presenting acidic soil Ph and higher annual pluviometry) and also because of the use of aluminum containers to store the honeys [61].

The determination of the mineral content in honey may not only be interesting from a nutritional perspective—where it is known to possess essential minerals required for the proper body growth and functions—but it also allows for quality control and/or anthropogenic biomonitoring, in relation to high levels of certain minerals that can be dangerous and cause toxicity [3,5,59,62].

The Biobío Region showed the biggest relevant differences (P, Mg, and Zn) compared to the rest of the territories under study, which could be related to the fact that the Biobío Region marks the transition between the temperate dry climates of Central Chile and the temperate rainy climates that develop south of the Biobío River.

## 4. Relation between Color, Mineral Content, Botanical Composition, and Classification of the Honey

A principal component analysis (PCA) was performed to assess whether there is a relation between color, and mineral and botanical composition, which showed that the botanical composition of the honeys did not explain the variation of the other variables analyzed. Therefore, a PCA was performed only with the color and mineral composition data. Thusly, a PCA model was generated with four useful principal components, since these are the ones that presented intrinsic values greater than 1, which combined explain 68.2% of the original information, as shown in Table 7. The Table 8 shows the contribution of each component and the correlation between the different variables.

The principal component analysis could not explain the variability among the variables, perhaps because—although the honeys are different in their mineral composition—they are not so variable as to be clearly differentiated within the sample.

**Figure 3:**
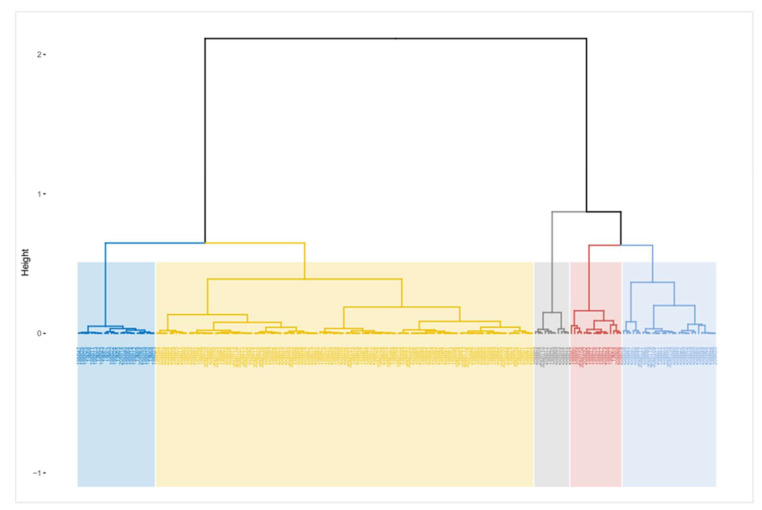
Cluster dendrogram.

Finally, a cluster analysis was performed among the variables with the objective of studying groups within the honeys analyzed to see if it is possible to classify them with objective variables. The cluster obtained (Figure 3) shows the formation of five quality groups within the total number of honey samples analyzed.

The blue group is represented by honeys from the province of Chiloé (75%), by TPV honeys (17%), and by honeys from the Los Ríos Region (only 4.2%). Additionally, said group is mainly made up by multifloral honeys (63%), Ulmo (20%), and—in smaller amounts—by species such as Fabaceae, Tineo, and Eucalyptus.

The yellow group is the largest one with 116 samples of honeys. Predominantly honeys from the TPV (31%), Los Ríos Region (22%), Osorno and Llanquihue Province and Chiloé Province all with (15.5%), with a low presence of honeys from the Araucanía and Biobío Regions. This group was dominated by multifloral (34%), Ulmo (30%), and Tineo (13%) honeys. Fabaceae, Tiaca, and Soapbark species were found in low proportions.

The grey group was the smallest one with only 5.5% of the honeys analyzed. Presence of honeys from the Province of Osorno and Llanquihue (45%), and the province of Chiloé (27.3%) were predominant. Additionally, multifloral honeys predominated (45%) as well as honeys from Ulmo and Lotera—both with an 18%.

The fourth group (red color) was mainly represented by the Province of Osorno and Llanquihue (38%), followed by the Los Ríos Region (31.3%), and—to a lesser extent—by the other territories analyzed. The presence of multifloral honeys (69%), Ulmo honeys (18.5%), and Fabaceae honeys (12.5%) stood out in this group.

The last group formed corresponds to the light-blue group. It is the second largest with 29 samples. Predominantly from Los Ríos Region (65.5%), TPV (27.6%), and the Biobío Region (20.7%), with a scarce presence of the other territories. This group was mainly made up by multifloral (41%) and Ulmo (17.4%) honeys.

The cluster analysis shows that the honeys from the Los Ríos and Los Lagos Regions (including all their provinces) could be differentiated from the honeys from the other territories analyzed, for which it is necessary to increase the number of samples of honeys from the Biobío and Araucanía Regions.

## 5. Conclusions

In Chile’s central-southern region, monofloral honeys predominate (56%)—with species such as *Eucryphia cordifolia* (Ulmo), leguminous plants (*Lotus* sp. and *Melilotus* sp.), *Weinmannia trichosperma* (Tineo), and *Quillaja saponaria Mol.* (Soapbark). While 42% corresponds to multifloral honeys, only 2% of the honeys were classified as bifloral, with *Eucryphia cordifolia* (Ulmo), *Weinmannia trichosperma* (Tineo), and *Caldcluvia paniculata* (Tiaca) being the predominant species.

From a physicochemical perspective, the honeys from Chile’s central-southern region comply with the ranges stipulated by both the national and international legislations, standing out as honeys of excellent nutritional quality, freshness, harvested at optimum maturity, with no unwanted fermentation. Honey color showed great variability (22–101 mm Pfund). However, medium-to-dark colors predominated, with extra-light amber and light amber categories being predominant in Chile’s central-southern region.

The cluster analysis reveals an important variation within the groups, which is a reflection of the different types of honeys produced in each territory, allowing a first classification by color. These results could be complemented with chemometric analysis for the identification and classification of the origin of the honeys produced in Chile’s central-southern region.

Considering the current market trend for safe and one-of-a-kind quality products, differentiating the honeys produced in Chile according to their botanical origin and nutritional composition—especially those coming from territories recognized for their high endemism, native flora biodiversity, climate, and low human intervention—signifies a great potential to add value to honey production.

## Figures and Tables

**Table 1:** Distribution and number of honey samples analyzed.

Location	Location on the Map	District	*n*
Biobío Region	1	Santa Juana, San Pedro de la paz, Concepción, Cañete, alto Bío Bío.	14
La Araucanía Region	2	Pucón, Nueva Imperial, Cholchol, Villarrica, Temuco, Cautín, Malleco.	17
Los Ríos Region	3	Panguipulli, Los Lagos, Lanco, Chol Chol, Loncoche, Pichihuilma, Futrono, San José de la Mariquina, Casa Blanca, Llifen, Pilfitrana.	46
* Osorno and Llanquihue Province (Los Lagos Region)	4	Pichidamas, Trumao, Puyehue, Frutillar, San Pablo, Tegualda, Fresia, Pichilcura, Purranque, Río Negro, Osorno.	29
Chiloé Province (Los Lagos Region)	5	Puqueldón, Chonchi, Ancud, Castro, Queilen, Quemchi, Dalcahue, Cahuala, Los Arroyos, Quellón, Curaco de Vélez.	41
Territorio Patagonia Verde (TPV) (Los Lagos Region)	6	** Cochamó, Hualaihué, Hornopirén, Futaleufú, Chaitén, Chaiguaco, Alto Puelo, Sotomo, San Ignacio de Loyola.	53

* For this investigation the Osorno and Llanquihue Provinces were considered as one sampling zone. ** Cochamo belongs to the Llanquihue province but for this study it was considered the territorial distribution according to the current regional politic-administrative distribution. (Figure 1b).

**Table 2:** USDA (United States Department of Agriculture) Universal Color Scale (mm Pfund).

Color	Range (mm Pfund)
Water white	0–8
Extra white	9–17
White	18–34
Extra light amber	35–50
Light amber	51–85
Amber	86–114
Dark amber	>114

**Table 3:** Main botanical species found in the pollen profile of honeys studied by territory (% of pollen).

Territory	Species	Mean ± S.D
Biobío Region (*n* = 14)	* *Quillaja saponaria Mol.* (Soapbark)	31.1 ± 25.5
	** *Castanea sativa* (Sweet chestnut)	15.5 ± 19.0
	* *Azara petiolaris* (Corcolén)	10.8 ± 8.7
	** *Rubus ulmifolius* (Elmleaf blackberry)	8.4 ± 8.1
La Araucanía Region (*n* = 17)	* *Weinmannia trichosperma* (Tineo)	33.9 ± 28.6
	* *Quillaja saponaria Mol.* (Soapbark)	24.9 ± 13.3
	** *Castanea sativa* (Sweet chestnut)	13.5 ± 17.7
	** *Rubus ulmifolius* (Elmleaf blackberry)	10.5 ± 6.9
Los Ríos Region (*n* = 46)	* *Weinmannia trichosperma* (Tineo)	32.9 ± 28.9
	* *Eucryphia cordifolia* (Ulmo)	30.8 ± 34.2
	* *Caldcluvia paniculata* (Tiaca)	14.8 ± 16.4
	** *Lotus* sp.*, Melilotus* sp. (Fabaceae)	14.3 ± 22.7
Osorno and Llanquihue Province (Los Lagos Region) (*n* = 29)	** *Lotus* sp.*, Melilotus* sp. (Fabaceae)	38.3 ± 27.3
	* *Eucryphia cordifolia* (Ulmo)	31.4 ± 35.7
	* *Weinmannia trichosperma* (Tineo)	8.4 ± 15.3
	* *Myrtaceae* (Luma or Arrayán)	7.1 ± 7.4
Chiloé Province (Los Lagos Region) (*n* = 41)	* *Eucryphia cordifolia* (Ulmo)	30.7 ± 27.8
	** *Lotus* sp.*, Melilotus* sp. (Fabaceae)	19.0 ± 17.5
	* *Caldcluvia paniculata* (Tiaca)	18.9 ± 24.5
	** *Eucalyptus globulus* (Eucalyptus)	12.2 ± 17.6
TPV (Los Lagos Region) (*n* = 53)	* *Eucryphia cordifolia* (Ulmo)	43.1 ± 24.0
	** *Lotus* sp.*, Melilotus* sp. (Fabaceae)	15.1 ± 21.5
	** *Brassica rapa* (Field mustard)	14.3 ± 13.1
	* *Weinmannia trichosperma* (Tineo)	13.7 ± 14.3

Mean ± standard deviation of the pollen, * Native species, ** Exotic species, TPV (Patagonia Verde Territory).

**Table 4:** Classification and typification of the honey in relation to its percentage of predominant pollen distributed by territory (according to NCh 2981:2005) [11].

	Bíobío Region	La Araucanía Region	Los Ríos Region	Osorno and Lanquihue Province (Los Lagos Region)	Chiloé Province (Los Lagos Region)	TPV (Los Lagos Region)	Total
Sweet Chestnut Monofloral Honey	1	-	-	-	-	-	1
Fabaceae Monofloral Honey	-	-	4	12	4	2	22
Soapbark Monofloral Honey	3	-	-	-	-	-	3
Tiaca Monofloral Honey	-	-	2	-	4	-	6
Tineo Monofloral Honey	1	3	14	1	-	3	22
Ulmo Monofloral Honey	0	1	13	9	12	20	55
Fruit trees Monofloral Honey	-	-	-	-	1	-	1
Eucalyptus Monofloral Honey	-	-	-	-	1	-	1
Raps Monofloral Honey	-	-	-	1	-	-	1
Tiaca-Tineo Bifloral Honey	-	-	2	-	-	-	2
Tiaca-Ulmo Bifloral Honey	-	-	1	-	-	1	2
Multifloral Honey	9	13	10	6	19	27	84

**Table 5:** Chemical composition of honeys produced in Chile’s central southern region.

	* Humedad (%)			Color (Pfnd)			HMF (mg/k)			Diastasa (DN)				Protein (%)				Ash (%)		
	Mean S.D.	Min	Max	Mean S.D.	Min	Max	Mean S.D.	Min	Max	Mean S.D.	Min	Max	Mean S.D.		Min	Max	Mean S.D.		Min	Max
Biobío Region	18.1 ^ab^ ± 0.9	17	19.9	73.9 ^de^ ± 14.3	46	96.3	3.8 ^b^ ± 5.1	0	18.4	19.6 ^c^ ± 8.2	7	40	0.36 ^ab^ ± 0.1		0.3	0.5	0.25 ^ab^ ± 0.1		0.1	0.5
La Araucanía Region	17.4 ^a^ ± 0.9	16	19.7	73.6 ^e^ ± 11.4	64	91.0	3.4 ^b^ ± 2.7	0	9.7	17.5 ^c^ ± 3.7	11	27	0.44 ^c^ ± 0.1		0.3	0.6	0.25 ^ab^ ± 0.1		0.1	0.5
Los Ríos Region	17.9 ^ab^ ± 1.1	16	21.7	65.7 ^cd^ ± 11.5	45	100.7	3.4 ^b^ ± 4.7	0	29.9	13.7 ^b^ ± 6.6	5	32	0.41 ^b^ ± 0.2		0.2	1.2	0.25 ^ab^ ± 0.1		0.1	0.5
** Osorno and Llanquihue Province	17.7 ^ab^ ± 1.1	15.5	19.2	62.1 ^c^ ± 12.6	27	83.0	1.7 ^b^ ± 1.2	0	4.6	20.5 ^c^ ± 4.5	12	29.1	0.36 ^ab^ ± 0.1		0.2	0.5	0.22 ^a^ ± 0.1		0.1	0.5
** Chiloé Province	18.4 ^b^ ± 1.0	16.5	20.8	43.7 ^a^ ± 10.7	22.3	67.0	1.0 ^a^ ± 1.3	0	5.2	12.9 ^b^ ± 4.9	5	28.5	0.38 ^b^ ± 0.1		0.2	0.6	0.31 ^b^ ± 0.1		0.2	0.7
** TPV	17.8 ^ab^ ± 1.2	15.4	21.4	55.4 ^b^ ± 13.1	30	82.7	2.2 ^b^ ± 2.5	0	11	10.9 ^a^ ± 5.1	2	26	0.31 ^a^ ± 0.1		0.2	0.5	0.30 ^b^ ± 0.1		0.1	0.8
Valor *p*	0.033			<0.001			<0.001			<0.001				<0.001				0.002		

Mean ± standard deviation of the evaluated samples, different superscripts within columns represent a significant difference (*p* < 0.05). * Statistic analysis using ANOVA TPV: Territorio Patagonia Verde. ** Territories belonging to the Los Lagos Region.

**Table 6:** Mineral content of honeys produced in Chile’s central-southern region.

	Bíobío Region	La Araucanía Region	Los Ríos Region	Osorno and Llanquihue Province *	Chiloé Province *	TPV *	FAO Reference values ** (ppm)	Value *p*
P (ppm)	110.0 ^c^ ± 57.9	67.2 ^ab^ ± 20.3	72.8 ^b^ ± 33.3	67.8 ^bc^ ± 17.5	54.2 ^a^ ± 16.1	62.9 ^a^ ± 33.0	20–150	<0.001
Ca (ppm)	71.4 ^ab^ ± 29.1	65.8 ^ab^ ± 31.0	70.4 ^ab^ ± 24.7	88.3 ^b^ ± 42.6	83.2 ^b^ ± 38.8	60.5 ^a^ ± 23.4	30–310	0.019
Mg (ppm)	30.0 ^b^ ± 17.1	19.8 ^b^ ± 9.1	23.2 ^b^ ± 11.3	22.3 ^ab^ ± 11.0	17.5 ^a^ ± 4.9	20.6 ^ab^ ± 7.9	7–130	0.047
Na (ppm)	88.8 ^a^ ± 46.0	103.7 ^ab^ ± 44.6	125.3 ^b^ ± 44.6	174.6 ^c^ ± 76.1	182.8 ^c^ ± 66.6	113.0 ^ab^ ± 43.6	16–170	<0.001
K (ppm)	1184.4 ^ab^ ± 899.0	960.9 ^ab^ ± 495.5	1060.7 ^ab^ ± 413.9	905.1 ^a^ ± 589.1	1261.9 ^b^ ± 387.8	1167.4 ^b^ ± 319.0	400–35,000	0.014
Al (ppm)	27.5 ± 22.5	35.8 ± 29.8	39.7 ± 29.5	47.8 ± 46.2	38.0 ± 37.2	31.3 ± 21.0	0.1–24	0.501
Zn (ppm)	1.4 ^c^ ± 0.8	0.7 ^abc^ ± 0.3	1.1 ^bc^ ± 1.0	0.6 ^a^ ± 0.3	0.7 ^a^ ± 0.4	0.9 ^ab^ ± 1.0	0.5–20	0.0018
Mn (ppm)	2.8 ^b^ ± 1.9	2.3 ^b^ ± 2.2	2.3 ^b^ ± 2.7	2.8 ^b^ ± 2.3	1.4 ^a^ ± 1.1	2.1 ^b^ ± 1.3	0.2–20	0.001
Fe (ppm)	4.5 ± 2.3	3.5 ± 2.3	3.7 ± 1.8	4.1 ± 2.6	3.4 ± 2.1	3.0 ± 1.6	0.3–40	0.151
Cu (ppm)	0.8 ± 0.5	0.6 ± 0.4	0.8 ± 0.6	0.7 ± 0.5	1.0 ± 0.7	0.7 ± 0.6	0.2–0.6	0.445

Mean ± standard deviation of the evaluated samples. Different superscripts within rows represent a significant difference (*p* < 0.05) * Territories belonging to the Los Lagos Region. ** Reference values of mineral content reported by [58].

**Table 7:** Principal component analysis (Intrinsic value, variance ratio, cumulated variance) for the variables color and mineral composition of honeys produced in Chile’s central-southern region.

	PC1	PC2	PC3	PC4	PC5	PC6	PC7	PC8	PC9	PC10	PC11
Standard Deviation	1.902	1.317	10.683	1.004	0.859	0.829	0.764	0.726	0.675	0.566	0.424
Proportion of Variance	0.39	0.157	0.103	0.091	0.067	0.062	0.053	0.047	0.041	0.029	0.016
Cumulative Proportion	0.329	0.486	0.590	0.682	0.749	0.811	0.864	0.912	0.954	0.983	1.000

**Table 8:** Correlation matrix of principal component analysis for the variables color and mineral composition of honeys produced in Chile’s central-southern region.

	CP1	CP2	CP3	CP4
Color	0.220	−0.368	0.310	−0.394
P	0.344	−0.409	−0.045	0.143
Ca	0.301	0.334	−0.249	−0.179
Mg	0.455	−0.169	0.025	0.041
Na	0.144	0.432	−0.439	−0.071
K	0.318	0.199	0.348	0.311
Al	0.304	0.375	0.372	−0.140
Zn	0.222	−0.286	−0.223	0.557
Mn	0.163	−0.292	−0.385	−0.554
Fe	0.383	0.107	0.207	−0.149
Cu	0.308	0.092	−0.380	0.165

## Data Availability

The data presented in this study are available on request from the corresponding author.
